# The Knee-Fix study: study protocol for a randomised controlled trial evaluating cemented and cementless components in total knee arthroplasty

**DOI:** 10.1186/s13063-022-06974-7

**Published:** 2022-12-20

**Authors:** Mei Lin Tay, Nina Zeng, Sherina Holland, Ali Bayan, Bill J. Farrington, Rupert van Rooyen, Rob Sharp, Robert S. J. Elliott, Matthew L. Walker, Simon W. Young

**Affiliations:** 1 Department of Orthopaedic Surgery, North Shore Hospital, Auckland, New Zealand; 2 Department of Surgery, University of Auckland, Auckland, New Zealand

**Keywords:** Total knee arthroplasty, Cement fixation, Cementless, Aseptic loosening, Radiolucencies

## Abstract

**Background:**

Total knee arthroplasty (TKA) is an effective procedure for patients with a variety of knee conditions. The main cause of aseptic TKA failure is implant loosening, which has been linked to poor cement mantle quality. Cementless components were introduced to offer better longer-term biological fixation through osseointegration; however, early designs led to increased rate of revision due to a lack of initial press-fit and bony ingrowth. Newer highly porous metal designs may alleviate this issue but randomised data of fully uncemented TKA (tibial, femoral, patella) is lacking. The aim of the Knee-Fix study is to investigate the long-term implant survival and patient outcomes of fully uncemented compared with cemented fixation in TKA. Our study hypothesis was that uncemented TKA would be as clinically reliable and durable as the gold-standard cemented TKA.

**Methods:**

The Knee-Fix study is a two-arm, single-blinded, non-inferiority randomised controlled trial with 160 patients in each arm and follow-up at 6 weeks, 6 months, 12 months, 24 months, 5 years and 10 years. The primary outcome of interest is implant fixation, which will be measured by assessment of postoperative progressive radiolucencies with the Knee Society Total Knee Arthroplasty Roentgenographic Evaluation and Scoring System. Secondary outcome measures are patient-reported outcomes, measured using Oxford Knee Score (OKS), International Knee Society System (IKSS), Forgotten Joint Score-12 (FJS-12), EuroQol (EQ-5D-5L), VAS Pain, Patient Satisfaction Score and Net Promoter Score.

**Discussion:**

While cemented fixation remains the gold standard, a growing proportion of TKA are now implanted cementless. Highly porous metal cementless components for TKA can offer several benefits including potentially improved biological fixation; however, long-term outcomes need further investigation. This prospective study will help discern long-term differences between the two techniques.

**Trial registration:**

Australian New Zealand Clinical Trials Registry ACTRN12616001624471. Registered trial name: Knee-Fix study (Cemented vs Uncemented Total Knee Replacement). Registered on 24 November 2016.

## Administrative information

Note: the numbers in curly brackets in this protocol refer to SPIRIT checklist item numbers. The order of the items has been modified to group similar items (see http://www.equator-network.org/reporting-guidelines/spirit-2013-statement-defining-standard-protocol-items-for-clinical-trials/).

**Table:** 

Title {1}	The Knee-Fix study: study protocol for a randomised controlled trial evaluating cemented vs. cementless components in total knee arthroplasty
Trial registration {2a and 2b}.	Australian New Zealand Clinical Trials Registry Identifier ACTRN12616001624471. Registered trial name: Knee-Fix study (Cemented vs Uncemented Total Knee Replacement). Registered on November 24, 2016.
Protocol version {3}	Protocol version 1.2, May 2019
Funding {4}	This study is funded by the Orthopaedic Education and Research Fund: Three Harbours Health Foundation (Waitemata District Health Board, New Zealand). Stryker (New Zealand) provided research assistant funding support for facilitating follow-up visits.
Author details {5a}	^1^Department of Orthopaedic Surgery, North Shore Hospital, Auckland, New Zealand ^2^Department of Surgery, University of Auckland, Auckland, New Zealand
Name and contact information for the trial sponsor {5b}	North Shore Hospital, Takapuna, Auckland 0740, New Zealand.
Role of sponsor {5c}	The study sponsor is not involved in study design, collection, management, analysis, and interpretation of data; writing of the report; and any decisions to submit the report for publication.

## Introduction

### Background and rationale {6a}

Total knee arthroplasty (TKA) is an effective procedure for patients with a range of knee conditions, including osteoarthritis, osteonecrosis, rheumatoid arthritis, and arthritis resulting from trauma. This procedure has shown successful outcomes for more than 90% of patients over 20 years [1, 2]. As there is increasing need for TKA, and patient groups are younger with higher expectations of function [3, 4], ensuring implant longevity is important.

The leading cause of aseptic TKA failure is loosening of the implant, which can account for up to 30% of all failures [5, 6]. This mode of failure is linked to poor cement mantle quality and can be identified from the radiographic presence of progressive radiolucent lines [7]. In total hip arthroplasty, the use of cementless components has led to improved fixation and reduced incidence of aseptic loosening, including in younger patients [8]. Therefore, it is anticipated that use of cementless components for TKA may also lower the incidence of loosening and improve implant survival. There are other benefits offered by cementless components, including better bone stock preservation [9], avoidance of cement debris and decreased operating time [10]. In the longer term, the osseointegration provided by cementless components may also outlast cemented fixation, as bone-cement interfaces can wear away over time [11].

The Stryker Triathlon Tritanium® implant that will be used in this study involves complex 3D-printing layering of titanium powder on the surface of baseplates and metal-backed patella [12], creating a highly porous three-dimensional interface. The trabecular titanium coating on the proximal surface of the tibial baseplate is extended to part of the pegs and keels to ensure close contact of the bone-implant interface, allowing for minor inconsistencies with bone resection and morphology. The trabecular titanium metal-backed patella is of a monoblock design. The uncemented implants can also be cemented in situ, which provides the surgeon with intraoperative flexibility once bone quality is assessed. Early reports of the femoral and tibial components of this design show good survivorship in both registry data and peer-reviewed publications [6, 13–15]; however, some studies on previous designs of cementless patella components tend to have higher early revision rates, suggesting a lack of initial press-fit and bony ingrowth [16, 17]. The higher coefficient of friction and rapid ingrowth seen with newer highly porous metal designs may alleviate this issue [11, 18]. Randomised data on fully uncemented (tibial, femoral, patella) in comparison to fully cemented TKA is lacking.

Although cemented fixation remains the gold standard [19], a growing proportion of TKA are now implanted cementless [6, 20]. The aim of the Knee-Fix study is to investigate the long-term implant survival and patient outcomes of fully uncemented compared with cemented fixation in TKA. Our study hypothesis was that uncemented TKA would be as clinically reliable and durable as the gold-standard cemented TKA.

## Objectives {7}

The objectives of the Knee-Fix study are to investigate:

1. If TKA with fully uncemented highly porous metal components are as clinically reliable as TKA with standard cemented components. Outcome measures are health status, functional outcome measures, complications and reoperations.
2. If TKA with fully uncemented highly porous metal components are as durable as TKA with standard cemented components (as judged by survivorship and assessment of progressive radiolucencies).

## Trial design {8}

The Knee-Fix study is a prospective, single-centre, single-blinded, non-inferiority randomised controlled trial. Eligible participants are enrolled into one of two arms: (1) uncemented group: treatment with total knee arthroplasty using a Stryker Triathlon cementless implant with cementless patellar resurfacing, or (2) cemented group: treatment with total knee arthroplasty using a Stryker Triathlon cemented implant with cemented patellar resurfacing.

## Methods: participants, interventions and outcomes

### Study setting {9}

The study site is North Shore Hospital, Takapuna, Auckland 0740, New Zealand. All patients will have surgery and inpatient stays at North Shore Hospital or the Elective Surgery Center, Takapuna, Auckland 0740, New Zealand. Recruitment and follow-up will be at North Shore Hospital and/or Waitakere Hospital, Henderson, Auckland 0610, New Zealand.

### Eligibility criteria {10}

#### Inclusion criteria

Patients must (1) be male or non-pregnant female between 40 and 75 years old, (2) be eligible for cruciate retaining knee replacement, (3) have a primary diagnosis of osteoarthritis, (4) have intact collateral ligaments, (5) have signed the study-specific informed consent document and (6) be willing and able to comply with specified pre-operative and post-operative clinical and radiographic evaluations.

#### Exclusion criteria

Patients (1) have had total, unicompartmental reconstruction or fusion of the affected joint; (2) have previous osteotomy around the knee; (3) have a body mass index (BMI) ≥ 41; (4) have a deformity requiring use of stems, wedges or augments with the Triathlon Total Knee System; (5) have a varus/valgus malalignment ≥ 20° (relative to mechanical axis); (6) have a fixed flexion deformity ≥ 20°; (7) have a neuromuscular or neurosensory deficiency; (8) have a systemic or metabolic disorder leading to progressive bone deterioration or poor bone quality; (9) are immunologically suppressed or receiving steroids in excess of normal physiological requirements; (10) have a cognitive impairment, an intellectual disability or a mental illness; (11) are not undergoing patellar resurfacing; or (12) are having simultaneous bilateral TKA.

### Who will take informed consent? {26a}

The investigators or delegated research assistants will carry out informed consent according to Good Clinical Practice (GCP). This includes informing potential candidates about the purpose and duration of the study, as well as explaining study-specific evaluations, risks and potential benefits that may result from being in the study. Participants are free to refuse participation, decline or withdraw from the study at any time without compromising their medical care. Participants will be given time to read, understand and, if agreeable, sign the study-specific Participant Consent Form prior to study enrolment.

### Additional consent provisions for collection and use of participant data and biological specimens {26b}

Biological specimens are not collected for this study and collection of participant data is incorporated into the consent process for participation listed in “Who will take informed consent? {26a}” section.

### Interventions

#### Explanation for the choice of comparators {6b}

While cemented fixation remains the gold standard for TKA, the optimal mode of fixation is still debatable. Research comparing the outcomes of cementless vs cemented TKA shows contrasting results [21–23]. In recent years, the use of cementless components has been proposed to offer better biological fixation and improved survivorship due to the improved design features [6, 14, 15]. This prospective randomised controlled trial will help discern long-term differences between the two techniques. As uncemented implants can offer some clinical benefits [13], we plan to conduct non-inferiority analyses, that is, use of fully uncemented TKA is considered acceptable if it is not worse than the gold-standard fully cemented TKA.

#### Intervention description {11a}

The seven consultant surgeons participating in the study will each have performed over 100 TKAs, will be experienced with navigation equipment and will have performed at least five cementless cases in a pilot phase before the study begins. Both study groups will receive primary TKA using the Triathlon Primary Total Knee System (all cruciate-retaining) and Stryker PrecisioN Knee Navigation System [24] according to the Triathlon Knee System Surgical Protocol [25]; however, one group will receive cemented implantation and the other group will receive cementless implantation. A tourniquet will be used for cementation in all cases. Patients will be enrolled to one of the two intervention groups at the time of surgery using allocation concealment.

#### Criteria for discontinuing or modifying allocated interventions {11b}

Surgeons may choose to modify interventions intraoperatively if they feel intra-operative findings mean alternative implants are required, such as the use of stems, augments or additional constraint. Additionally, if inadequate primary fixation (press fit) is obtained with uncemented fixation, the surgeon may choose to cement one or more components. These will be recorded as protocol deviations, and both intention-to-treat and per-protocol analyses will be performed.

#### Strategies to improve adherence to interventions {11c}

The research assistant will prepare randomised concealed allocation of intervention using sealed envelopes, which will be assigned to consecutive patients in consecutive order. Patients are blinded to their intervention group throughout the duration of the study, and surgeons will only be unblinded at time of surgery. All participants will be classified as ‘intent to treat’ and those who did not receive the allocated intervention will continue to be followed up for outcome and safety purposes.

#### Relevant concomitant care permitted or prohibited during the trial {11d}

Appropriate post-operative care will be provided according to the preference of the treating physician and all patients will undergo inpatient and outpatient postoperative rehabilitation programmes according to standard of care.

#### Provisions for post-trial care {30}

All participants will receive standard-of-care follow-up and medical advice from their treating physicians after completion of the study. If adverse outcomes occur as a result of study participation, participants will still receive the same appropriate per usual health care practice as those not involved in the study. TKA patients at our institution undergo a rehabilitation programme, including group knee classes, as standard of care. Physiotherapists carrying out rehabilitation are blinded to the study intervention.

### Outcomes {12}

The main reason for TKA failure is aseptic loosening; therefore, the primary outcome of interest is implant fixation, which will be measured by assessment of postoperative progressive radiolucencies. Standard anterior-posterior (AP) and medio-lateral (ML) X-rays of the study limb will be obtained pre-operatively and post-operatively at 6–12 weeks, and 2, 5 and 10 years post-operatively (Table 1). Radiographic assessment will be performed following the Knee Society Total Knee Arthroplasty Roentgenographic Evaluation and Scoring System [26], which will produce a single score for each component. All radiographs will be measured by one investigator to ensure consistency. The score at the 5-year follow-up will be the primary outcome of interest; however, the scores at other follow-ups will also be measured and presented.

**Table 1:** Patient evaluation schedule for Knee-Fix study: an RCT evaluating cemented vs. cementless total knee arthroplasty

	Study period						
	Enrolment	Allocation	Post-allocation				Close-out
Timepoint	***−t***_***1***_ (−2 months)	0	***t***_***1***_ (6–12 weeks)	***t***_***2***_ (12 months)	***t***_***3***_ (24 months)	***t***_***4***_ (5 years)	***t***_***5***_ (10 years)
**Enrolment:**							
**Eligibility screen**	X						
**Informed consent**	X						
***Demographics, medical history***	X						
**Allocation**		X					
**Intervention:**							
***Cemented TKA***		X					
***Cementless TKA***		X					
**Assessments:**							
***AP, ML and Skyline X-rays***	X	X	X^a^		X	X	X
***Oxford Knee Score***	X		X	X	X	X	X
***International Knee Society System***	X				X	X	X
***Forgotten Joint Score-12***					X	X	X
***EQ-5D-5L***	X				X	X	X
***VAS Pain***	X		X	X	X	X	X
***Patient Satisfaction score***				X	X	X	X
***Net Promoter Score***				X	X	X	X

*AP* Anterior-posterior, *EQ-5D-5L *Euroqol 5 dimensions, *ML* Medio-lateral, *TKA *total knee arthroplasty, *VAS* Visual analogue scale

^a^6–12 week X-rays only for patients with inadequate immediate post-op images

Secondary outcomes will be measured using validated questionnaires: Oxford Knee Score (OKS) [27], International Knee Society System (IKSS) [28], Forgotten Joint Score-12 (FJS-12) [29], EuroQol (EQ-5D-5L) [30], VAS Pain [31], Patient Satisfaction Score and Net Promoter Score. These questionnaires will provide a quantitative measure of patient pain, function, health and satisfaction. They will be completed by patients preoperatively and at 6–12 weeks, and 1, 2, 5 and 10 years post-operatively (Table 1).

### Participant timeline {13}

The proposed patient evaluation schedule is presented in Table 1.

### Sample size {14}

The main outcome measure is the incidence of radiolucencies using plain X-rays. To calculate sample size, we used a non-inferiority test; that is, the uncemented-fixation technique is acceptable if it is not worse than the standard cemented TKA technique based on the incidence of radiographic lucencies. Power calculations were based on estimates from a previous RCT, where 11% of cemented TKA and 2% of uncemented TKA were found to have progressive radiolucencies [21]. Using a power of 80%, 95% confidence level and a non-inferiority limit of 9%, 150 patients are needed in each group. An additional 10 patients per group (5%) will be recruited to allow for loss to follow-up, bringing the target to 160 patients per group (total of 320 patients). The estimated loss to follow-up of 5% is lower than other studies, however is based on previous experience of minimal loss to follow-up of our RCT patient cohorts [32]. Every individual in New Zealand accessing health systems is assigned a national unique health identifier, which allows for comprehensive capture of patient contact details, clinical notes and radiographs, including those who have moved to a different area.

### Recruitment {15}

If participants meet eligibility criteria, they will be randomised to either the cemented group or the uncemented group, with no other factors of influence.

## Assignment of interventions: allocation

### Sequence generation {16a}

A block randomisation process will be used for this study. A central randomisation list will be computer generated by a consultant statistician at a 1:1 ratio. This method will ensure allocation of patients into the two groups with an equal sample size over time.

### Concealment mechanism {16b}

Randomisation envelopes marked with a randomisation number and containing the corresponding group allocation (cemented or uncemented) will be prepared by an independent member of staff at the institution’s research centre. Patients will be assigned to allocation sequentially according to randomisation number.

### Implementation {16c}

On the day of surgery, the study investigators will locate and open a randomisation envelope in sequence. The randomisation number will be used as the patient’s study ID number and noted in the Case Report Form.

## Assignment of interventions: blinding

### Who will be blinded {17a}

This is a single-blinded trial as the investigators (surgeons) and research staff cannot be blinded to the intervention to deliver treatment and monitor intervention delivery. In order to minimise bias, patients will be blinded to their group allocations for the study period. Pre- and post-operative X-rays will be reviewed by a reviewer blinded to the identity of the patient and of the surgeon.

### Procedure for unblinding if needed {17b}

Patients will be unblinded to the intervention at the end of the trial, unless there is a medical reason to do so prior to the end of the study.

## Data collection and management

### Plans for assessment and collection of outcomes {18a}

Source data will be captured via paper forms that will be either mailed out to participants or filled out at clinic visits. Study windows for data collection have been calculated for each timepoint as follows: Pre-Op, within 2 months before surgery, 6–12 weeks (± 2 weeks), 12 months (± 2 months), 24 months (± 3 months), 5 years (± 6 months) and 10 years (± 6 months).

### Plans to promote participant retention and complete follow-up {18b}

Reasonable effort will be made to ensure a complete dataset, including phone calls for missed questions and to participants who fail to respond after 14 days, and at least three attempts to contact participants at study visits. An X-ray appointment will be made, including for participants who have relocated to ensure radiographs are available for study analysis. Attempts will continue to be made to contact participants who did not complete a study visit for each subsequent study visit.

### Data management {19}

Source data will be collected on paper forms and entered into the electronic study database by trained research staff. The paper case report forms will be stored securely at the investigator site. Archiving will be undertaken in accordance with GCP guidelines. Verification of the data from source documents will also be conducted.

### Confidentiality {27}

Only the investigators and data management team involved with this study will have access to the study database. Participant data will be stored on a secure network only accessible via hospital computers. All data files will be password protected. Only deidentified data will be used for analysis, publication or dissemination. All research staff working on this study will first undergo standard privacy training and signed confidentiality agreements with the hospital.

### Plans for collection, laboratory evaluation and storage of biological specimens for genetic or molecular analysis in this trial/future use {33}

Not applicable as there is no planned collection of biological specimens for this study.

## Statistical methods

### Statistical methods for primary and secondary outcomes {20a}

The primary outcome measure is measurement of progressive radiolucencies graded using the Knee Society Roentgenographic Score (KSRS) [26] at 5 years. The proportion of patients in each group scored at 4 or above (indicating possible progression) will be compared for each compartment using Fisher’s exact test. Descriptive statistics of the KSRS will also be presented at the other follow-ups.

The secondary outcome measures are the OKS, IKSS, FJS, EQ-5D-5L, VAS Pain, Patient Satisfaction and Net Promoter Scores. Results will be summarised as mean and standard deviation for continuous variables, or frequencies and percentages for categorical variables. The differences calculated will be both absolute and as change scores (change from baseline) at each timepoint. Longitudinal outcomes will be analysed using linear mixed effects models, with adjustment for repeated measurements and including important baseline surgery and patient demographics (age, gender, BMI, American Society of Anesthesiologists (ASA) score, smoking status) as covariates. Pairwise comparisons will be compared with *t*-tests (normally distributed) or Mann-Whitney (non-parametric). Categorical data will be compared using Fisher’s exact test or chi-squared tests. A *p*-value of <0.05 will be considered significant. Adverse events will be tabulated separately and reviewed for any commonalities. Revision surgery data from the study will be compared to the revision rates reported in the National Joint Registry [6] at 2 years, 5 years and 10 years.

### Interim analyses {21b}

Formal interim analyses will be performed at completion of the 2-year, 5-year and 10-year visits. Results of these analyses will be used for dissemination of study results.

### Methods for additional analyses (e.g. subgroup analyses) {20b}

No additional subgroup analyses are planned.

### Methods in analysis to handle protocol non-adherence and any statistical methods to handle missing data {20c}

Non-adherence to randomisation will be analysed both as intention-to-treat and per-protocol. Missing data will be imputed according to instrument guidelines if necessary.

### Plans to give access to the full protocol, participant level-data and statistical code {31c}

The full protocol is presented here. Collected data will not be made available to the public. A lay summary of findings will be provided to the participants who requested this during the consenting process after completion of the study.

## Oversight and monitoring

### Composition of the coordinating centre and trial steering committee {5d}

The coordinating centre is North Shore Hospital, Takapuna, Auckland 0740, New Zealand. The trial steering committee includes the principal investigator, a research coordinator and research assistants involved with study coordination. The committee will meet on a monthly basis.

### Composition of the data monitoring committee, its role and reporting structure {21a}

A Data Safety Committee comprising of the principal investigator, a consultant orthopaedic surgeon independent of the study, a statistical advisor and a patient advocate will be formed to review the data and any adverse event forms.

### Adverse event reporting and harms {22}

All adverse events will be documented in an electronic site log. All serious adverse events will be documented on case report forms by the research assistant and reviewed by the primary investigator. Date of occurrence, description, severity, relationship to study device, treatment and date of resolution will be captured. The investigator will comply with the applicable regulatory requirements related to the reporting of serious unexpected adverse device reactions to the regulatory authority (MedSafe NZ) and the national ethics committee.

### Frequency and plans for auditing trial conduct {23}

There are no plans for formal auditing of trial conduct; however, there will be a yearly report of trial progress and protocol deviations to the national ethics committee.

### Plans for communicating important protocol amendments to relevant parties (e.g. trial participants, ethical committees) {25}

Amendments to the protocol will be reported to the national ethics committee, and an application for approval submitted if significant amendments need to be made. Amendments will be communicated to study participants as necessary.

## Dissemination plans {31a}

Findings from this study will be disseminated through peer-reviewed publications and conferences by the study investigators. A lay summary of the findings will be shared with participants at the completion of the study.

## Discussion

Fully cementless TKA components offer promising benefits such as bone stock preservation [9], avoidance of cement debris, decreased operating time [10] and improved biological fixation [11]. However, older designs have been associated with higher early revision rates due to lack of bony ingrowth, particularly for the tibial and patellar components [16, 17]. Newer highly porous metal designs introduced with the aim of improving biological fixation have shown promising survivorship [6, 14, 15] but randomised data is lacking for fully cemented (tibia, femur, patella) TKA.

Based on reports involving earlier designs of cementless TKA components, there is a chance that patients may be at risk of being treated with a component that may be deemed inferior after further analysis; however, there is nothing a priori to suggest this. Patients will be carefully monitored during the study and interim analyses are planned after 2 years to ensure patient safety.

## Trial status

Protocol version 1.2, May 2019. The study began recruiting in September 2017. Decision was made to complete recruitment in March 2020 as a result of the unexpected impact of COVID-19 lockdowns on elective surgery volumes. Last patient visit for this 5-year study is anticipated to be in 2025.

## Data Availability

Identifiable data will only be made available to the investigators of the trial. Deidentified data can be shared upon request.

## Abbreviations

ANOVA: Analysis of variance
AP: Anterior-posterior
EQ-5D-5L: EuroQol 5 Dimension
FJS-12: Forgotten Joint Score-12
GCP: Good Clinical Practice
IKSS: International Knee Society System
ML: Medio-lateral
OKS: Oxford Knee Score
TKA: Total knee arthroplasty
VAS: Visual Analog Scale
